# Ovarian hyperstimulation syndrome in a frozen-thawed embryo transfer pregnancy: a rare case report

**DOI:** 10.1186/s12884-020-03014-7

**Published:** 2020-05-20

**Authors:** Lihua Yang, Rong Wang, Fang Wang, Feifeng Wang, Libo Zou

**Affiliations:** Department of Reproductive Medicine, Jinhua People’s Hospital, Jinhua, 321000 Zhejiang China

**Keywords:** Ovarian hyperstimulation syndrome, Frozen-thawed embryo transfer, Dichorionic diamniotic triplet pregnancy, Multifetal pregnancy reduction

## Abstract

**Background:**

Ovarian hyperstimulation syndrome (OHSS) is an iatrogenic complication of ovarian stimulation. Prevention and early recognition of OHSS are important to ensure patient safety.

**Case presentation:**

In this case, we reported a patient who underwent controlled ovarian hyperstimulation (COH) and in vitro fertilization (IVF). All embryos were cryopreserved to reduce possible OHSS. However, OHSS still occurred after the patient had a frozen-thawed embryo transfer (FET) with hormone replacement therapy (HRT) and obtained a dichorionic diamniotic triplet pregnancy. After multifetal pregnancy reduction (MFPR) and supportive treatment, all the symptoms regressed.

**Conclusions:**

Prompt recognition of OHSS, especially in patients who have no history of ovulation induction and fresh embryo transfer, is very important. Multiple pregnancies may lead to severe OHSS because of the high level of human chorionic gonadotropin (hCG) in the early stages. We suggest that a single embryo transfer may be necessary and beneficial for patients.

## Background

Ovarian hyperstimulation syndrome (OHSS) is an iatrogenic complication of ovarian stimulation, which may cause a life-threatening condition. Although the incidence of the severe form of OHSS is low, with reported values ranging from 0.2 to 1.2%, the syndrome remains a serious problem for infertility specialists due to its potentially fatal outcome [1]. OHSS, however, is extremely rare and can occur in normal spontaneous pregnancies [2–6]. Hence, we could not prevent OHSS even though some strategies were adopted, such as the GnRH agonist trigger instead of hCG and freezing all embryos. In 2014, one case reported OHSS following a thawed embryo transfer cycle [7]. Complications of spontaneous OHSS, such as ovarian torsion, are rare but should always be considered [8]. Here, we report a rare ovarian hyperstimulation case after frozen and thawed embryo transfer in a hormone replacement therapy (HRT) cycle.

## Case presentation

**Fig. 1:**
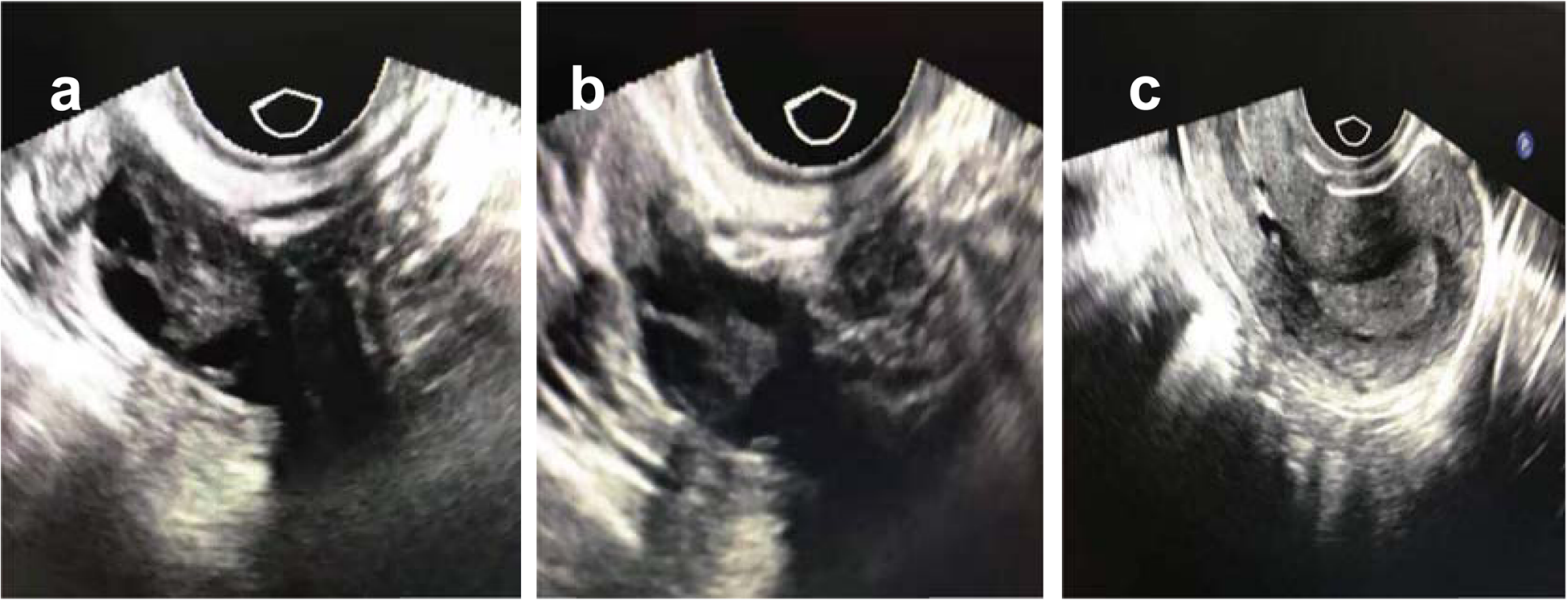
Transvaginal ultrasonography of the ovaries and uterus cavity on the day of embryo transfer. (**a** Left ovary **b** Right ovary **c** Uterus cavity)

**Fig. 2:**
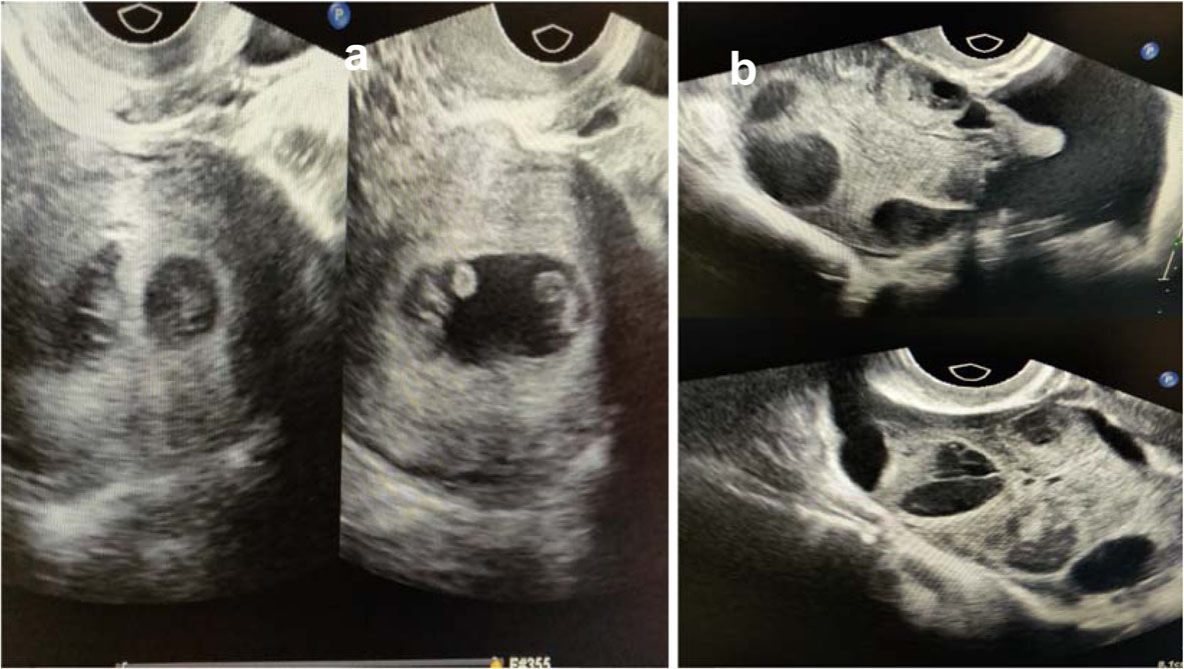
Two chorionic sacs were visualized by transvaginal ultrasound without any inter-twin membrane between two of the triplets. **b** Transvaginal ultrasonography of bilateral enlarged multicystic ovaries (Upper: Left ovary Lower: Right ovary)

A 30-year-old woman with secondary infertility was receiving assisted reproductive technology (ART) treatment at Jinhua People’s Hospital. She had been married for more than 2 years and underwent a one-sided salpingectomy because of ectopic pregnancy. The fallopian tube on the other side was blocked according to hysterosalpingography (HSG). Her height was 156 cm, her weight was 48 kg, and her body mass index (BMI) was 19.7 kg/m^2^. Her menstrual cycle was regular (28–30 days), thyroid-stimulating hormone (TSH) level was normal (2.23 μIU/ml), and the antral follicle count was 8–9/9–10 on the transvaginal ultrasound scan. Neither other illnesses nor other operations were found. She came to our clinic for help and underwent IVF. We used a long-acting protocol to stimulate her ovaries. On day 21 of her previous cycle, GnRH agonist (0.8 mg; GnRHa; Diphereline, Ipsen, France) treatment began. And on day 3 of her next cycle, ovulation induction was performed with 75 IU follicle-stimulating hormone (FSH; Puregon, Organon, Oss, Netherlands) and 75 IU human menopausal gonadotropin (hMG; Pergonal, Serono, Aubonne, Italy). The stimulation lasted for 10 days and a total of 250 μg of recombinant human choriogonadotropin (r-hCG; Ovidrel, Serono, Aubonne, Italy) was administered to trigger ovulation. On the trigger day, the levels of FSH, E2 and P4 were 10.77 mIU/ml, 4601 pg/ml and 1.00 ng/ml respectively. Total of 20 oocytes were aspirated from 21 follicles, and 19 oocytes were matured. We performed regular IVF fertilization, and 13 oocytes were fertilized and cultured. According to her E2 level and the oocytes received, we thought she had a high risk of OHSS and decided to cancel the fresh embryo transfer. On day 5 of oocyte retrieval, 2 blastocyst embryos (4 BC, 4 AC) were formed and cryopreserved. No OHSS developed. After 6 weeks, we performed frozen-thawed embryo transfer and hormone replacement therapy in which progynova and progesterone were given for endometrial preparation. Progynova began on day 3 of her cycle when the E2 and P4 levels were 26 pg/ml and 0.20 ng/ml respectively. The progesterone was added on day 16 when the E2 and P4 levels were 150 pg/ml and 0.10 ng/ml respectively. On day 21, we performed the embryo transfer. The images of the ovaries and uterus cavity on the day of embryo transfer is shown in Fig. 1(a-c). On day 9 after the two blastocyst transfers, the hCG level was 248.7 mIU/ml. On day 23 after the transfer, we performed the transvaginal ultrasound scan and detected a dichorionic diamniotic pregnancy. The patient came back to our clinic at 6 weeks after the blastocysts transfer with complaints of lower abdominal pain and progressive swelling of the abdomen along with nausea, oliguria, and dyspnea. Her abdomen was distended due to ascites. Transvaginal sonography was performed and showed a dichorionic diamniotic triplet pregnancy of 8 weeks (Fig. 2a), with bilateral enlarged multicystic ovaries (Fig. 2b). Her white blood cell (WBC) count was 9.56 × 10^6^/ml, neutrophils were 81.3%, hematocrit (HCT) was 41.2%, fibrinogen was 7.3 g/l, and D-dimer was 1360 μg/l. Renal function and liver function test results were within normal limits. Serum hCG showed a very high level > 225,000 mIU/ml, AFP was 24.90 U, and CA125 was 65.16 U. USG-guided aspiration of the cyst and ascitic fluid sent for cytology were negative for malignancy. Thus, the diagnosis of severe OHSS with viable intrauterine dizygotic dichorionic diamniotic triplet pregnancy was made. Considering the relative risks and severe hyperstimulation symptoms, the patient chose the multifetal pregnancy reduction (MFPR) operation (from three to one) and selected to reduce the monoamniotic twins. The patient was given intravenous (IV) fluid supplement to maintain her blood volume. One month later, her symptoms gradually improved, and the ascites gradually subsided. On July 18th, 2019, the patient successfully gave birth to one healthy baby by cesarean section at week 39 of pregnancy.

## Discussion and conclusions

In this case, we reported that OHSS occurred in a patient with frozen and thawed embryo transfer of the HRT cycle in association with a dizygotic dichorionic diamniotic triplet pregnancy. OHSS is a common, complex iatrogenic complication after human-assisted technology. The pathologic mechanism of OHSS is still unclear. One generally accepted hypothesis is that an increase in the permeability of capillaries causes fluid leakage into the third lacuna, thus causing the systematic hemodynamic changes [9]. The main influence factors of OHSS include age, BMI, allergies, etiology of infertility, types of stimulatory drugs, gonadotrophin dosage, exogenous hCG, response to ovulation stimulation, follicular number, luteal supplementation, and pregnancy rate [10]. Prevention and early recognition of OHSS are important to ensure patient safety. In this case, despite low-dose gonadotropin stimulation was used, 20 oocytes were collected. To decrease the risk of severe OHSS, all the qualified embryos were cryopreserved and the blastocysts transfer was postponed. When we planned to perform FET, the hormone level returned to normal, and both ovaries were quiet. By accident, OHSS may be associated with a spontaneous ovulatory cycle, as in some cases. However, in our case, there was no evidence to show that any follicles developed according to ultrasonography scan images during the hormone replacement cycle. OHSS in spontaneous pregnancies is a very rare event, and etiopathogenesis is not well studied. De Leener et al. [11] proposed a classification based on three different pathophysiological mechanisms responsible for the occurrence of spontaneous OHSS syndrome. Type I was associated with FSH receptor gene mutation, which might cause recurrent spontaneous OHSS. Type II was secondary to high levels of hCG in multiple gestations, which was the most frequent. And Type III was related to hypothyroidism. In our case, this patient had a high level of hCG and triplet gestation and presented with severe OHSS with signs of hemoconcentration, oliguria, and tense ascites. Her condition was treated successfully using MFPR and IV-fluid therapy [12]. Besides, we noticed that the blastocyst formation rate was low (only 2 blastocysts formed on day 5 after fertilization from 13 zygotes). All 13 embryos were scored grade IV on day 3 after fertilization, probably because of her low-quality eggs or unexplained reasons. Grade IV embryos are not qualified for direct transplantation due to the low development potential. The blastocyst formation rate for low-quality embryos was 14.1–28.4% according to the data published in 2017 [13].

Currently, the increasing use of ART has increased the rate of triplet pregnancies [14]. Although some policies have been made to limit the number of transferred embryos to two, there is still a relative proportion of triplets accompanied with mixed chorionicity as one of the two transferred embryos may split [15]. In general, monoamniotic multiple pregnancies are rare and occur when one fertilized ovum divides between days 7 and 13 after fertilization [16]. The detection of chorionicity is essential. In our case, two blastocysts were transferred into the patient’s uterus. We did not recognize a dichorionic diamniotic triplet pregnancy at the first scan, which maybe because it was too small to distinguish. A dichorionic diamniotic triplet pregnancy was diagnosed at approximately 8 gestational weeks. Data have shown that multiple pregnancy has a high risk of severe prematurity, while MFPR can reduce the risk of preterm delivery and severe OHSS [17, 18]. Besides, Multiple pregnancy with monochorionic twins is associated with additional complications because of unique angioarchitecture in the placental bed [19]. A cohort study showed that multifetal pregnancy reduction from three to one notably decreases the risk of severe preterm delivery [20].

When referring to patients who have symptoms of OHSS and at the same time have no history of ovulation induction, clinicians might make the wrong decision, such as laparotomy, because of suspicion of ovarian carcinoma [21]. Our patient developed severe OHSS after frozen-thawed embryo transfer. After MFPR and supportive treatment, all the symptoms recovered. The induction factor of OHSS in our case may be the multiple pregnancy. Thus, we can expect a single blastocyst transfer will reduce the incidence of OHSS and benefit patients.

## Data Availability

All data are available in the manuscript.

## Abbreviations

OHSS: Ovarian hyperstimulation syndrome
COH: Controlled ovarian hyperstimulation
IVF: In vitro fertilization
FET: Frozen-thawed embryo transfer
HRT: Hormone replacement therapy
MFPR: Multifetal pregnancy reduction
hCG: Human chorionic gonadotropin
ART: Assisted reproductive technology
HSG: Hysterosalpingography (HSG)
BMI: Body mass index
TSH: Thyroid-stimulating hormone
IV: Intravenous
WBC: White blood cells
HCT: Hematocrit
